# Vitamines and Domestic Education

**Published:** 1920-07-31

**Authors:** 


### FOOD VALUES AND COMMON-SENSE.

### II. Vitamines and Domestic Education.

In article I. we endeavoured to explain that, as far as we know, there are three important accessory food factors, or vitamines—viz., fat soluble "A" (anti-rachitic), water soluble "B" (anti-neuritic), and an anti-scorbutic factor. The first two we found were necessary for growth, and they all possess not only the power of preventing certain definite diseases, but of promoting and maintaining bodily health in many ways. None of these substances have been isolated, and their chemical composition is unknown. They exist in various foods in extremely minute quantities and out of all proportion to the power they possess, and their presence is only known by feeding animals on foods known to be chemically pure and free from vitamines, allowing the animals to develop the characteristic symptoms of ill-health due to the absence of each of the vitamines in turn, and then restoring them to health by adding known quantities of certain food extracts and constituents known to contain the vitamine in question, and finally noting the amount of these required to restore the animal to normal health and growth.

### Common Food Articles.

In this way a very large range of common articles of food have been examined, and although, for reasons given above, exact quantities in each food cannot be given, still a very good idea can be obtained by an examination of the tables supplied in the Research Committee's Report referred to in article I., where an attempt has been made to give a quantitative estimate by placing the signs O, +, ++, and +++ after each article of food examined and under the heading of the three vitamines. Another interesting and important fact to remember is that these accessory factors can only be manufactured or, to use the modern chemical term, "synthesised" by plants, and that we can obtain them in two ways, either direct from the plant which manufactures and contains them, or from the flesh of some herbivorous or other animal which obtains them either direct or indirect from the original source.

The next important question is, What happens to these accessory food factors through cooking, drying, and other forms of preservation? Taking first fat soluble "A," we find that it is gradually destroyed by heating at 100° C.—*i.e.*, ordinary boiling point—and that four hours at this temperature will render butter-fat no better than so much lard, which means that the vitamine has been completely destroyed. The practical deduction is here that milk, if heated up to or near boiling point, should be immediately cooled, and that fats which are stewed a long time lose much of their nutrient value. This applies also to tinned or canned meats, which are heated up much above boiling point.

As regards the water soluble "B" or anti-neuritic vitamine contained in such cereals, etc., as rice and wheat, this will stand cooking at 100° C.—*i.e.*, not only ordinary boiling, but baking where in the interior of the loaf the temperature does not rise above 100° C. Finally, it has been shown that the anti-scorbutic is the most sensitive of all, and is very largely destroyed by ordinary cooking and completely destroyed by drying. Cooking does *not* completely destroy them, else scurvy would be much more prevalent than it is; but dried fruit and vegetables are perfectly useless as anti-scorbutics—a point which has been demonstrated among ships' crews relying on them instead of fresh fruit and vegetables.

### Practical Housekeeping.

Having now considered the main characters and uses of vitamines, we come now to the practical application of our knowledge to the every-day catering of the housewife. The first matter to emphasise is that for growing children the fat and water soluble vitamines are absolutely essential and much more necessary than in the case of adults. The anti-scurvy vitamine is of course necessary, but the supply does not require to be quite so systematically administered. Taking first the fat soluble "A," we see, on looking at the list, that only three substances have three +s—viz., butter, cod-liver and other fish-liver oils, and egg-yolk. Unfortunately all these articles at the present time are very expensive and out of reach of the ordinary-class working mother, especially if she have a large, growing family, when the vitamine in question is so necessary; but on looking down the list we find plenty of animal fats with two +s. For instance, you have beef and mutton fat, fat fish such as herring, also several internal organs such as the liver, kidneys, and heart. Among cereals you have wholemeal bread, and vegetables such as fresh cabbage, lettuce, and spinach show two +s, also wholemilk cheese. Margarine made from vegetable fats, as it mostly is now, is perfectly useless, and should never take the place of ordinary beef or mutton fat. Bacon is not specially mentioned in the list, but presumably the fat would be classified as lard, which is quite useless from a vitamine point of view.

Coming next to the water soluble "B," or anti-neuritic factor, we find that rice with the germ heads the list with four +s, but it cannot be too often emphasised that *polished rice* is followed by an O, and is practically little better than so much starch. The next foods in order of merit with three +s are wheat germ, lentils, yeast, egg-yolk, ox-liver; dried peas have two +s, also haricot beans, and in this connection it is a great pity that more whole-meal bread is not eaten. It contains both fat soluble "A" and water soluble "B" in abundance, and from a nutritive point of view is much superior to white bread. Coming lastly to anti-scorbutics, these seem to be most abundant in raw vegetables and fruit. Fresh raw cabbage, raw swede juice, fresh lemon juice, and oranges head the list with three +s, but it may safely be said that all ordinary edible vegetables, especially when raw, contain anti-scorbutic vitamine.

One article of food specially valuable is milk, since it contains the one fat with the greatest amount of vitamine except cod-liver oil, that is, butter in a cheap and very digestible form, and this should form a staple article of diet for children during the ordinary school life. As the vital importance of these accessory food factors and the foods containing them cannot be known to the ordinary mother of the middle and working classes, it would be a good idea if sanitary authorities periodically issued a leaflet explaining briefly their nature and giving a list of the more common articles of food containing them.

The previous article appeared on June 19, p. 298.

